# The effects of MEPaL on oxidative stress and motor function in the rats affected by prenatal hypoxia

**DOI:** 10.1002/brb3.3539

**Published:** 2024-06-07

**Authors:** Hadis Nasri, Zohreh Ghotbeddin, Kaveh Rahimi, Mohammad Reza Tabandeh

**Affiliations:** ^1^ Department of Basic Sciences, Faculty of Veterinary Medicine Shahid Chamran University of Ahvaz Ahvaz Iran; ^2^ Department of Biochemistry and Molecular Biology, Faculty of Veterinary Medicine Shahid Chamran University of Ahvaz Ahvaz Iran; ^3^ Stem Cell and Transgenic Technology Research Center Shahid Chamran University of Ahvaz Ahvaz Iran

**Keywords:** balance, hypoxia, locomotor activity, methanolic extract of *Pistacia atlantica* leaves, oxidative stress, pregnancy, rat

## Abstract

**Background and objectives:**

Maternal hypoxia disrupts neural development and subsequently leads to cerebral palsy and epilepsy in newborns. Hypoxia plays a role in neurodegeneration by increasing oxidative stress. *Pistacia atlantica* is known as an important antioxidant, and its anti‐inflammatory and antioxidant effects have been shown in various studies. This study aims to investigate the effects of methanolic extract of *P. atlantica* leaves (MEPaLs) on the oxidative parameters in the serum of rats affected by maternal hypoxia.

**Material and methods:**

In this study, eight pregnant rats were used. The newborns were divided into four groups, including the control and the hypoxia groups, which are affected by maternal hypoxia, hypoxia + MEPaL 100 mg/kg, and hypoxia + MEPaL 150 mg/kg. MEPaL was injected (i.p) for 21 days into the neonatal rats after the lactation period. Hypoxia was induced by keeping pregnant rats in a hypoxic chamber with 7% oxygen and 93% nitrogen intensity for 3 h on the 20th day of pregnancy. Behavioral changes were measured using open‐field and rotarod tests. Finally, biomarkers of oxidative stress, nitric oxide (NO), glutathione (GSH), GSSG, TAS, TOS, and oxidative stress index (OSI) were measured in the experimental groups.

**Results:**

Behavioral results showed that the anxiety behavior in the hypoxia group increased, but the motor activity (moved distance and movement speed) decreased. Moreover, the amount of time spent maintaining balance on the rotarod rod was significantly decreased in the hypoxia group. The concentration of NO in the group of hypoxia + MEPaL 100 mg/kg showed a significant decrease, and MEPaL 100, and 150 mg/kg + hypoxia also increased the concentration of GSH and decreased GSSG. In addition, MEPaL100 and 150 mg/kg caused a significant increase in the ratio of GSH to GSSG and decreased OSI and total oxidant capacity.

**Conclusions:**

Oxidative stress increased in the rats affected by maternal hypoxia and may be the main mechanism for motor activity impairment and balance disturbance, whereas MELaL improved motor performance by decreasing oxidative stress.

## Graphical abstract

Display of groupings, protocols, and tests for assessing the effects of MEPaL on oxidative stress and motor function in the rats affected by prenatal hypoxia.

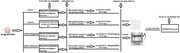

## 1 INTRODUCTION

Hypoxia is a disorder caused by insufficient blood and oxygen flow to the brain and is one of the main causes of seizures in infants (Rodríguez et al., 2020; Shetty, 2015). Neonatal hypoxia may cause complications such as learning and concentration deficits, hyperactivity, and memory impairment and subsequently lead to cerebral palsy and epilepsy in children (Marín‐Padilla, 1999; Piešová & Mojmír, 2020). Many infants are born with acute respiratory failure and experience hypoxia, and 20% and 50% of infants with hypoxic encephalopathy die in the first months of life, and more than 25% show permanent brain damage (Vannucci & Hagberg, 2004). Factors such as genetic mutation, inflammation, and ischemia/reperfusion cause the production of free radicals that affect the endogenous antioxidant defense mechanism and thus cause oxidative stress (Mo et al., 2022; Wang et al., 2022). Hypoxia and ischemia affect cognitive ability through blood circulation and brain metabolism disorders and cause significant neuropathological disorders. In addition, hypoxia can deplete tissue energy reserves and lead to acute biochemical events such as acidosis, glutamate toxicity, nitric oxide (NO) production, and oxidative stress in the central nervous system (Greco et al., 2020; Solevåg et al., 2019). Nerve cells are particularly vulnerable to free radicals because they contain high levels of unsaturated lipids and redox‐active transition metals that catalyze the formation of free radicals. Moreover, under oxidative stress conditions, dysfunctional mitochondria are unable to meet the high energy needs of nerve cells for their normal biochemical and physiological functions, so they become vulnerable to rapid cell death (Ashok et al., 2022; Barone et al., 2021). Today, antioxidant compounds are used to inhibit the formation of free radicals and subsequently stop oxidative stress, and attention has been paid to the role of these compounds in protecting the body against damage caused by oxidative stress (Akbari et al., 2022; Rana & Gautam, 2022). On the other hand, medicinal plants often have antioxidant activity, and, in addition to pain and inflammation, they are effective against some diseases such as neurological disorders, diabetes, and cancer that increase free radicals (Khazaei et al., 2021; Sangeetha et al., 2020). Methanolic extract of *Pistacia atlantica* leave (MEPaL) is a plant from Anacardiaceae family, which is also known as wild pistachio and grows mostly in the Zagros region of Iran (Mir‐Makhamad et al., 2022; Shahghobadi et al., 2019). *P. Atlantica* is known as an important antioxidant with its polyphenol and α‐pinene compounds, and its anti‐inflammatory and antioxidant effects have been shown in various studies (Saeedipour & Rafieirad, 2020; Shahghobadi et al., 2019). Various studies have supported the effects of *Pistacia* bioactive compounds for the treatment of central nervous system disorders such as Alzheimer's, Parkinson's, multiple sclerosis, cerebral ischemia, depression, and anxiety (Moeini et al., 2019; Yazdian‐Robati et al., 2023; Zahoor et al., 2018). In no study so far, the effect of MEPaL on the motor performance of rats following maternal hypoxia has been investigated, so the purpose of this study is to investigate the effect of MEPaL on motor function and oxidative stress in the rats affected by maternal hypoxia.

## 2 MATERIALS AND METHODS

### 2.1 Animals and grouping

In this study, eight pregnant Wistar rats were used. All procedures were designed and executed according to the Ethics Committee recommendations for laboratory animals at Shahid Chamran University of Ahvaz and complied with the NIH guidelines for the care and use of laboratory animals. After giving birth, the rats were maintained in a fully controlled standard animal facility (12:12‐h light:dark cycle at 22 ± 2°C and 55% ± 5% humidity). Vaginal smear analysis was used to detect the zero day of pregnancy. The presence of sperm cells or a cornified epithelial cell pattern suggested that fertilization was occurred and showed the zero day of pregnancy.

### 2.2 Animal model of hypoxic brain injury and drug treatments

The animals were randomly divided into four groups, including control, saline injection to newborns for 21 days, hypoxia‐exposed rats on day 20 of pregnancy + saline injection to newborns for 21 days, hypoxia‐exposed rats on day 20 of pregnancy + intraperitoneal injection of MEPaL at a dose of 100 mg/kg to newborns for 21 days, and hypoxia‐exposed rats on day 20 of pregnancy + intraperitoneal injection of MEPaL at a dose of 150 mg/kg to newborns for 21 days. Hypoxia was induced by keeping pregnant rats in a hypoxic chamber (7% oxygen and 93% nitrogen intensity) for 3 h. The leaves of MEPaL were prepared (Herbarium code of Faculty of Pharmacy, University of Tehran: PMP‐818), and the concentrated extract was homogenized in a certain volume of saline and then administered to the animals. Finally, on the 45th day of postnatal life, blood was taken from the newborns to measure oxidative stress parameters in the serum.

### 2.3 *Pistacia atlantica* extract preparation

*P. atlantica* fruit was prepared (Kurdistan, Iran) and dried using a medicinal plant dryer. The dried fruit is powdered. The obtained powder (100 g) was mixed in 1 L of ethanol 70%, and then the mixture was placed in a shaker incubator at a temperature of 25°C for 72 h. Then the mixture was filtered using Whatman Qualitative Filter Paper (Merck). In the next step, the extract was placed in a rotary (Buchi) at a temperature of 40°C, and ethanol was removed. The concentrated extract was turned into powder using a freeze dryer (Martin Christ) and kept at −20°C (Carneiro et al., 2022).

### 2.4 Behavioral tests

#### 2.4.1 Open‐field test

In the open‐field test, locomotor activity, including the frequency of rearing, movement velocity, and total distance traveled, was monitored and analyzed using a video‐tracking system for 10 min on the postnatal week (P44) in a black Plexiglas box (50 × 50 × 50 cm^3^) (Ghotbeddin et al., 2020).

#### 2.4.2 Rotarod test

The rotarod test was used to assess motor performance in the postnatal rats (P44). The animals were placed on the testing rod rotating at an initial speed of 5 rotations per minute (rpm). Then, the rod speed increased gradually to 45 rpm over 300 s. The time spent on the rod was recorded automatically for each animal. Each animal was initially given two opportunities for adaption to the device and then was tested three more times. The average time was calculated and considered for the analysis (Ghotbeddin et al., 2020).

### 2.5 Oxidative stress indicators

#### 2.5.1 Nitric oxide (NO)

The concentration of NO was evaluated by the ELISA method and according to the instructions of the manufacturer (Anasel), and the OD value was read at the wavelength of 570 nm and the concentration of NO was calculated. The values of different standard concentrations and the fitting line were drawn in Excel, and then the concentration was expressed as nmol/mL.

#### 2.5.2 GSH

Glutathione (GSH) assay was performed with DTNB following the standard method of Ellman. A volume of 2.3 mL of potassium phosphate buffer (0.2 M, pH 7.6) was taken in the cell, and 0.2 mL of aqueous solution (blood plasma) and 0.5 mL (0.001 M) of 5, 5‐Dithiobis, 2‐ were added to this solution. Nitrobenzoic acid (DTNB) was added to a buffer. The absorption of the reaction product was observed after 5 min at a wavelength of 412 nm with a microplate reader. The concentrations are expressed as nmol/mL.

#### 2.5.3 GSSG

The amount of GSSG was measured using Elman's reagent and Hu's method. To measure thiol groups, 2,2′‐dithiobis(5‐nitropyridine) was used. By reviving this reagent, thiol groups create a yellow complex that can be measured at 421 nm. The concentrations are expressed as nmol/mL.

#### 2.5.4 Assay of total antioxidant capacity (TAC) and total oxidant capacity (TOC)

TAC and TOC are abbreviations for total antioxidant capacity and total oxidant capacity, respectively. One of the new methods to assay and measure TAC and TOC in the serum of rat was based on the use of a novel chromogenic reagent, 3,3′,5,5′‐tetramethylbenzidine (TMB). This method used a colorimetric assay that measures the reduction of TMB by antioxidants and the oxidation of TMB by oxidants. The change in absorbance at 450 nm was proportional to the TAC and TOC values. To perform this method, we need the following reagents and equipment: TMB solution (10 mM in DMSO), acetate buffer (0.1 M, pH 4.5), hydrogen peroxide solution (30%), rat serum sample, spectrophotometer, microplate reader, microplate, pipettes, and tips. The procedure was as follows: Prepare the TMB working solution by diluting the TMB solution with acetate buffer to obtain an absorbance of 0.8 ± 0.02 at 450 nm. Prepare a standard curve of hydrogen peroxide by serially diluting the hydrogen peroxide solution with acetate buffer to obtain concentrations of 0, 10, 20, 40, 60, 80, and 100 μM. Pipette 200 μL of each hydrogen peroxide standard into a microplate well in triplicate. Pipette 200 μL of acetate buffer into a microplate well as a blank. Pipette 200 μL of rat serum sample into a microplate well in triplicate. Add 200 μL of TMB working solution to each well and mix well. Measure the absorbance at 450 nm immediately (*t*0) and after 10 min (*t*10) using a microplate reader. Calculate the TAC and TOC values using the following formulas (Wu et al., 2023):

$$\begin{array}{ccc} \mathrm{TAC}\left(\mathrm{mM}\right) & = & \left(\mathrm{TMB}\;t0-\mathrm{TMB}\;t10\right)/\left(H_{2}O_{2}\;t0-H_{2}O_{2}\;t10\right)\\ & & \times \;H_{2}O_{2}\;\mathrm{concentration} \end{array}$$

$$\begin{array}{ccc} \mathrm{TOC}\left(\mathrm{mM}\right) & = & \left(\mathrm{TMB}\;t10-\mathrm{TMB}\;t0\right)/\left(H_{2}O_{2}\;t10-H_{2}O_{2}\;t0\right)\\ & & \times \;H_{2}O_{2}\;\mathrm{concentration} \end{array}$$

#### 2.5.5 Oxidative stress index (OSI)

The ratio of the TOS level to the TAS level was accepted as the oxidative stress index (OSI). It is formulated as OSI (arbitrary unit) = TOS (mmol H_2_O_2_ equivalent/L)/TAS (mmol Trolox equivalent/L).

### 2.6 Statistical analysis

All data were presented as mean ± standard error (mean ± SEM). Normality of distributions was tested with the Shapiro–Wilk test. When the data were not normally distributed, we used a nonparametric Kruskal–Wallis test. The homogeneity of variance was checked with Levene's test. Welch's ANOVA was used for normal, different‐variance, and balanced data (i.e., same‐size samples), and Welch's has the most power and the lowest type I error rate. A classic ANOVA was run for an unequal sample size issue. The data were analyzed, and graphs were plotted using GraphPad Prism software version 9. The significance level was set at *p* < .05 (95% confidence intervals).

## 3 RESULTS

### 3.1 Open‐field test

**FIGURE 1:**
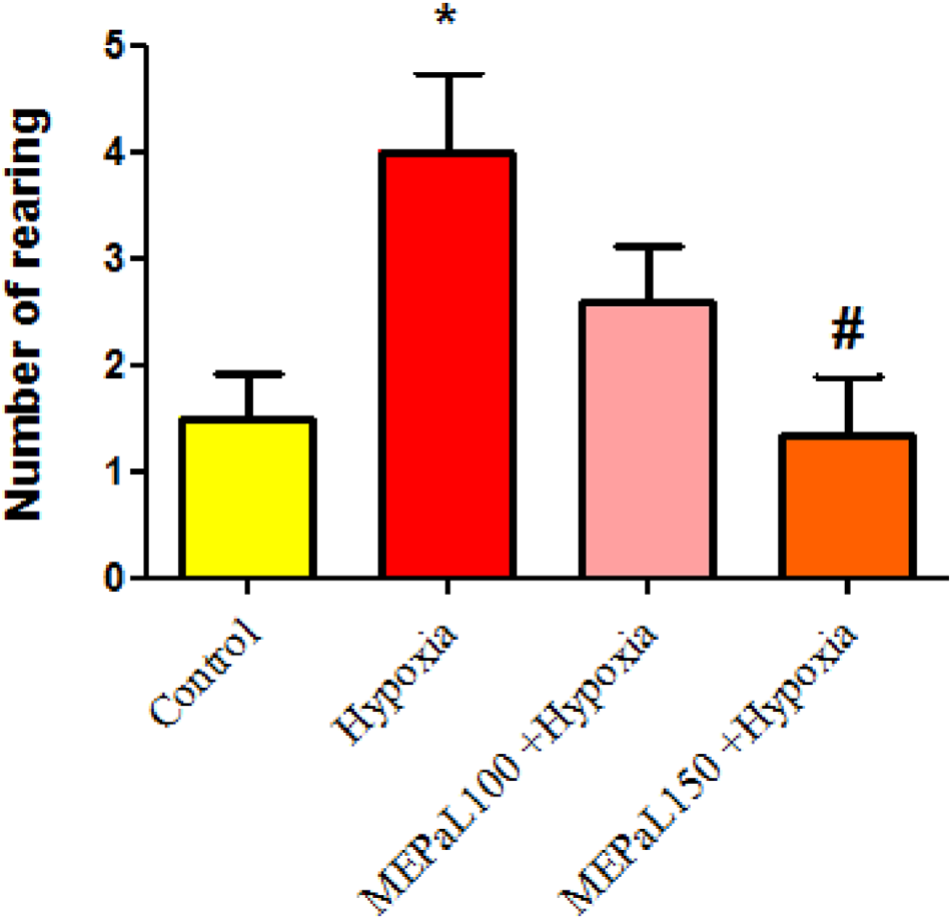
The number of rearing in the open‐field test. (*) indicates a significant difference in the hypoxia group compared to the control group (*p* < .05), (#) shows a significant difference between the treatment groups with the hypoxia (*n* = 8). All data are expressed in mean ± SEM.

**FIGURE 2:**
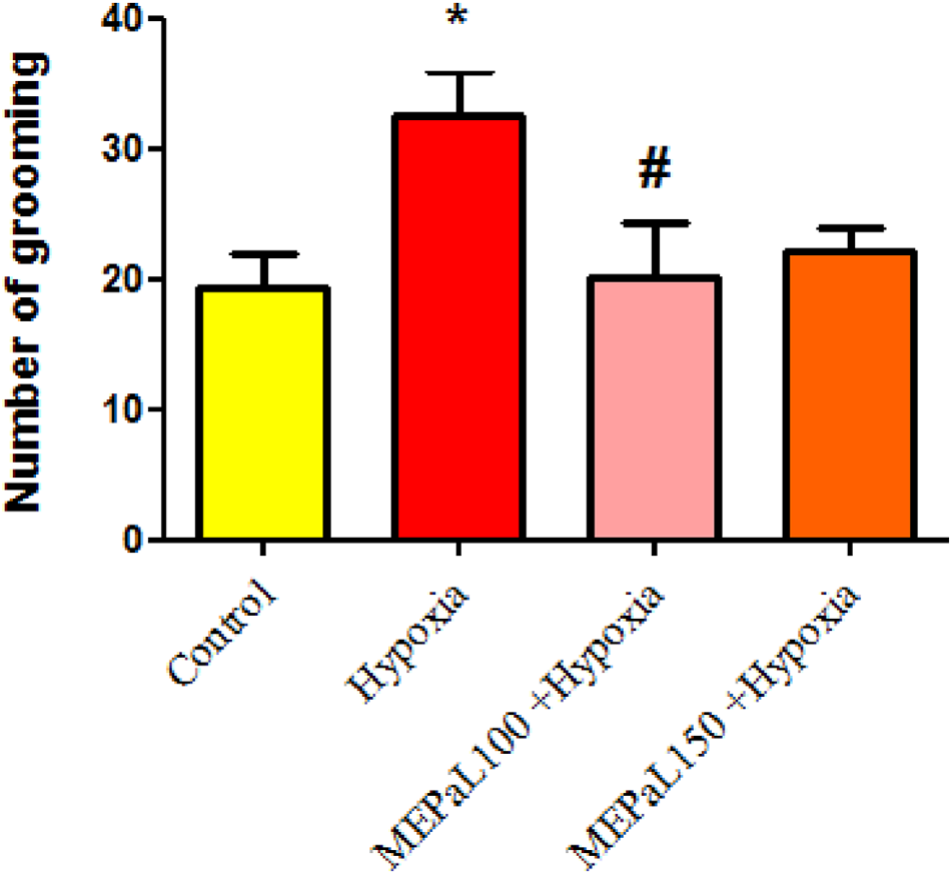
The number of grooming between groups in the open‐field test. (*) presents a significant difference between the hypoxia group and the control group (*p* < .05), (#) indicates a significant difference between the treatment groups with the hypoxia group (*n* = 8). All data are expressed in mean ± SEM.

**FIGURE 3:**
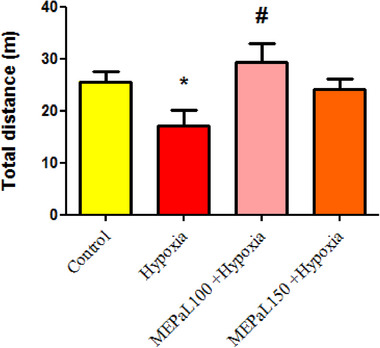
The average total distance in the open‐field test. (*) shows that a significant difference between the hypoxia and the control group at (*p* < .05), and (#) also shows a significant difference between the treatment groups with the hypoxia group. All data are expressed in mean ± SEM.

**FIGURE 4:**
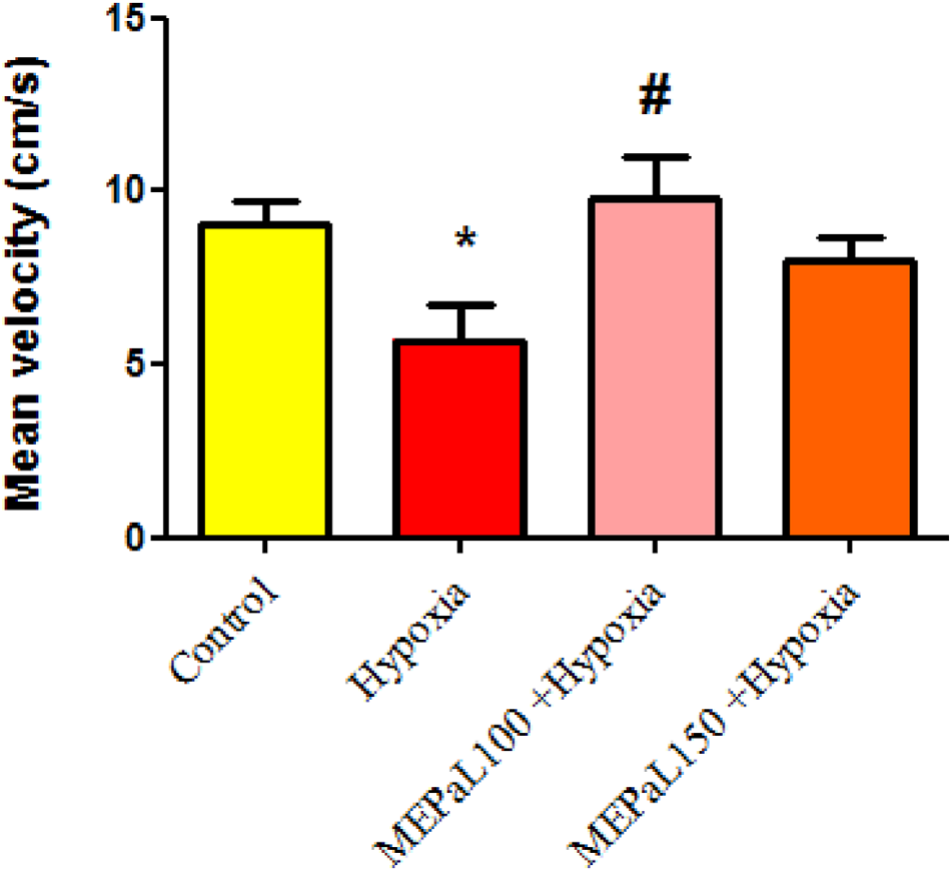
The average of movement velocity between groups. (*) indicates a significant difference in the hypoxia group compared to the control group (*p* < .05), (#) shows a significant difference between the treatment groups with the hypoxia group (*n* = 8). All data are expressed in mean ± SEM.

The results showed that the number of rearing in the hypoxia group increased significantly compared to the control group (*p* < .05), whereas, in the group exposed to hypoxia + MEPaL at a dose of 150 mg/kg, the number significantly decreased compared to the hypoxia group (Figure 1). The number of grooming in the hypoxia group increased significantly compared to the control group (*p* < .05) and in the group exposed to hypoxia + MEPaL at a dose of 100 mg/kg a significant decrease compared to the hypoxia group (*p* < .05) and no significant difference was observed between two dosages of MEPaL (Figure 2). Total distance and the average movement velocity in the hypoxia group decreased significantly compared to the control group (*p* < .05), whereas these two parameters were significantly increased in the hypoxia + MEPaL at a dose of 100 mg/kg group compared to the hypoxia group (Figures 3 and 4).

### 3.2 Rotarod test

**FIGURE 5:**
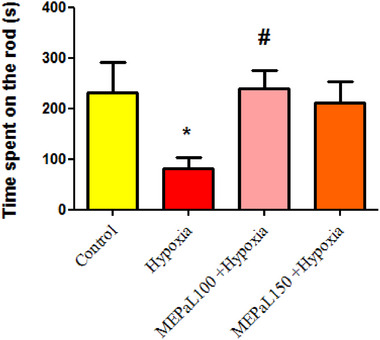
Motor performance in the rotarod test. (*) indicates a significant difference between hypoxia and the control group (*p* < .05), and (#) shows a difference between the treatment group and hypoxia (*n* = 8). All data are expressed in mean ± SEM.

The results of the rotarod test showed that the time spent maintaining balance on the bar in the hypoxia group was significantly reduced compared to the control group (*p* < .05) and significantly increased in the group exposed to hypoxia + MEPaL at a dose of 100 mg/kg compared to the hypoxia group (Figure 5).

### 3.3 Oxidative stress results

#### 3.3.1 Nitric oxide (NO)

**FIGURE 6:**
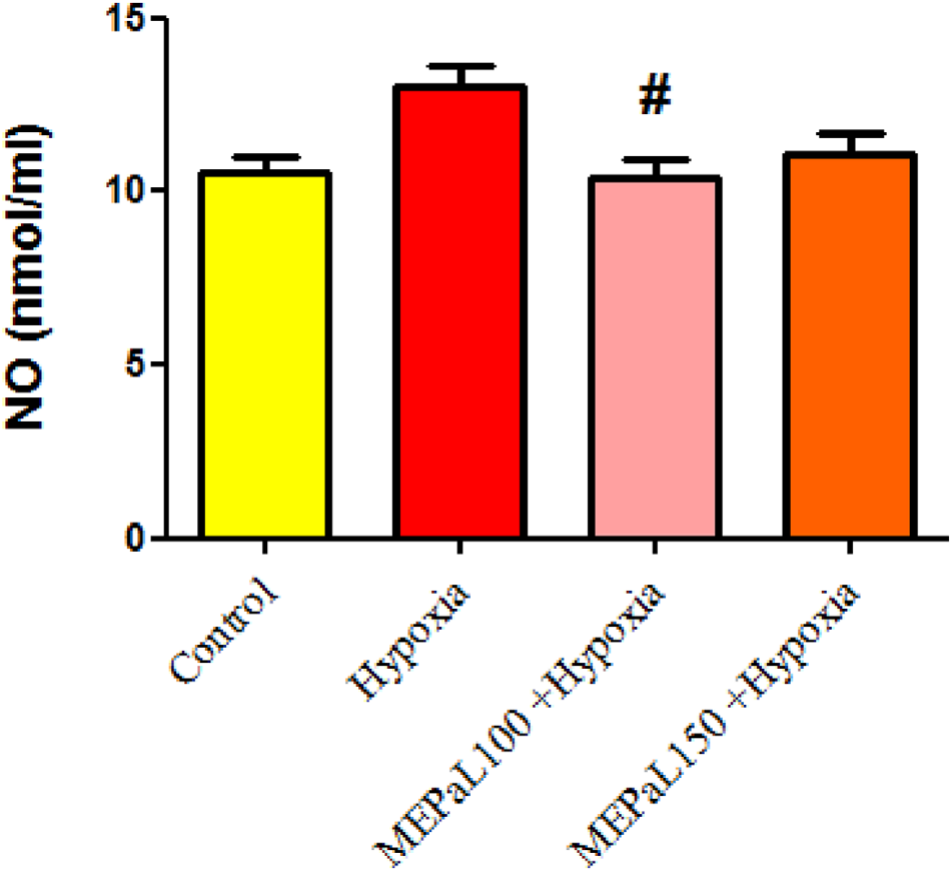
Comparison of nitric oxide (NO) concentration between groups. (#) indicates a significant difference between the treatment groups with the hypoxia group (*n* = 5). All data are expressed in mean ± SEM.

The results showed that there was no significant difference in the concentration of NO among the experimental groups, but it was significantly decreased in the group exposed to hypoxia + MEPaL at a dose of 100 mg/kg compared to the hypoxia group (*p* < .05) (Figure 6).

#### 3.3.2 Glutathione (GSH)

**FIGURE 7:**
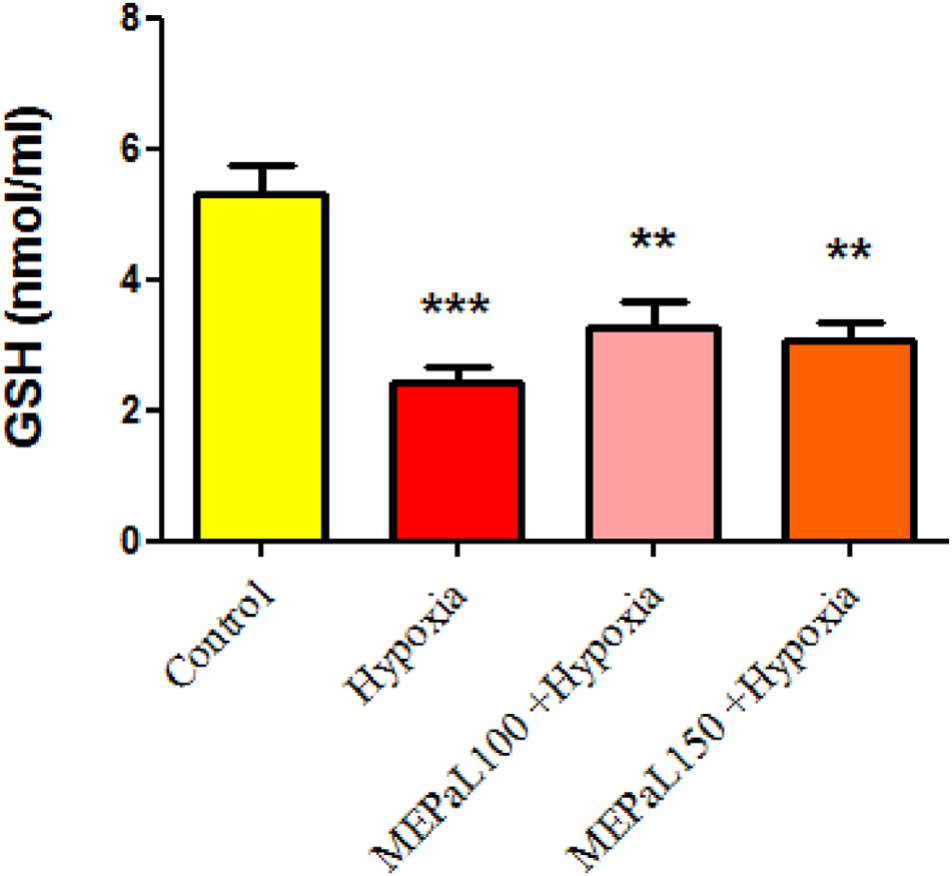
Comparison of glutathione (GSH) concentration between groups. (***) and (**) indicate a significant difference at the level of *p* < .001 and *p* < .01 between the experimental and the control groups (*n* = 5). All data are expressed in mean ± SEM.

The results showed that the concentration of GSH in the hypoxia group had a significant decrease compared to the control group (*p* < .001), and in the group exposed to hypoxia + MEPaL at a dose of 100 and 150 mg/kg (*p* < .01), it decreased significantly compared to the control group (Figure 7).

#### 3.3.3 Glutathione disulfide (GSSG)

**FIGURE 8:**
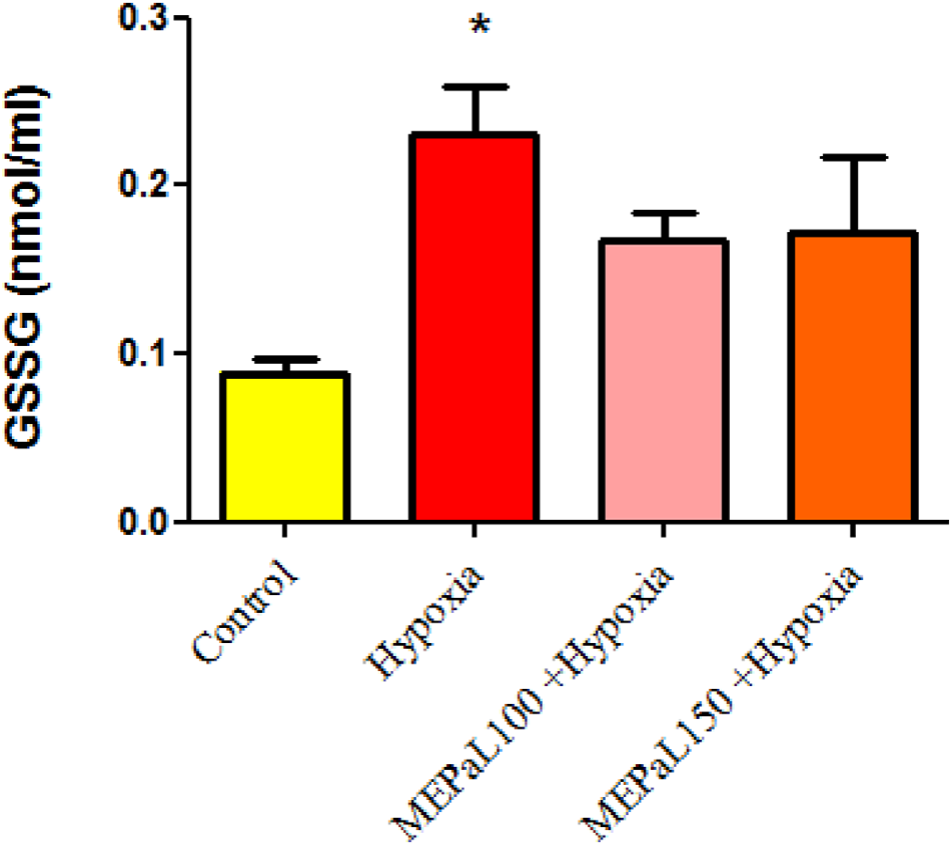
Comparison of GSSG concentration between groups. (*) shows a significant difference at the *p* < .05 level between the hypoxia and the control groups. All data are expressed in mean ± SEM.

**FIGURE 9:**
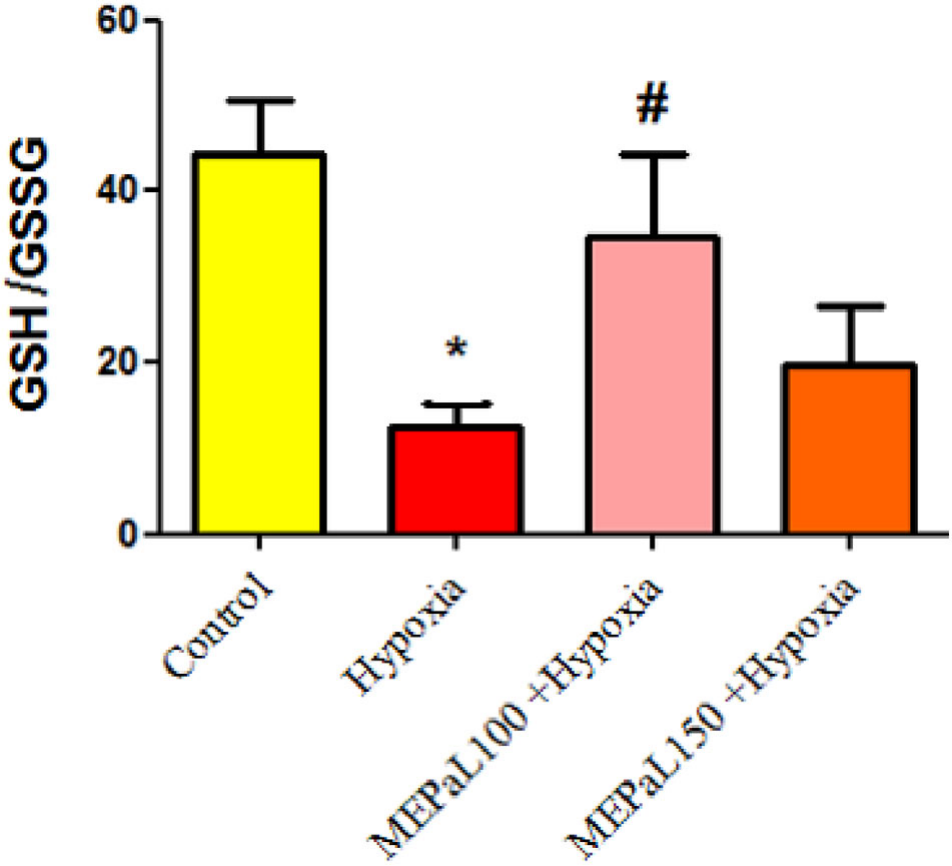
Comparison of glutathione (GSH) to GSSG ratio groups. (*)shows a significant difference at the level of *p* < .05 between the hypoxia and the control groups. (#) shows a significant difference between the treatment groups with the hypoxia group. All data are expressed in mean ± SEM.

The concentration of GSSG in the hypoxia group significantly increased in the hypoxia group compared to the control group (*p* < .05), and the difference between other experimental groups was not significant (Figure 8). According to the results, the ratio of GSH to GSSG in the hypoxia group had a significant decrease compared to the control group (*p* < .05), and it also showed a significant increase in the group exposed to hypoxia + MEPaL at a dose of 100 mg/kg compared to the hypoxia group (*p* < .05) (Figure 9).

#### 3.3.4 Total oxidant capacity (TOC)

**FIGURE 10:**
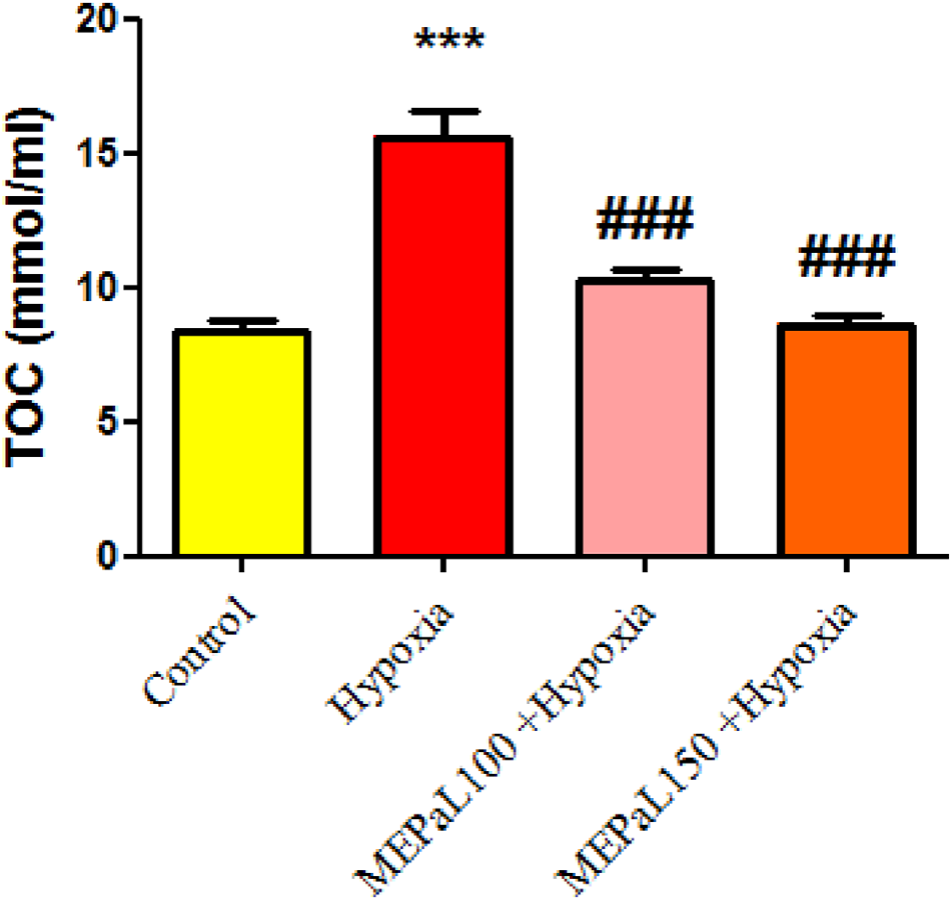
Comparison of total oxidant capacity (TOC) levels between groups. (***) indicates a significant difference at the level of *p* < .001 between the hypoxia and the control groups. (###) shows a significant difference between the treatment groups with the hypoxia group (*p* < .001) (*n* = 5). All data are represented in mean ± SEM.

TOC level in the hypoxia group increased significantly compared to the control group (*p* < .001), whereas it was decreased significantly in the group exposed to hypoxia + MEPaL at a dose of 100 and 150 mg/kg compared to the hypoxia group (*p* < .001) (Figure 10).

#### 3.3.5 Total antioxidant capacity (TAC)

**FIGURE 11:**
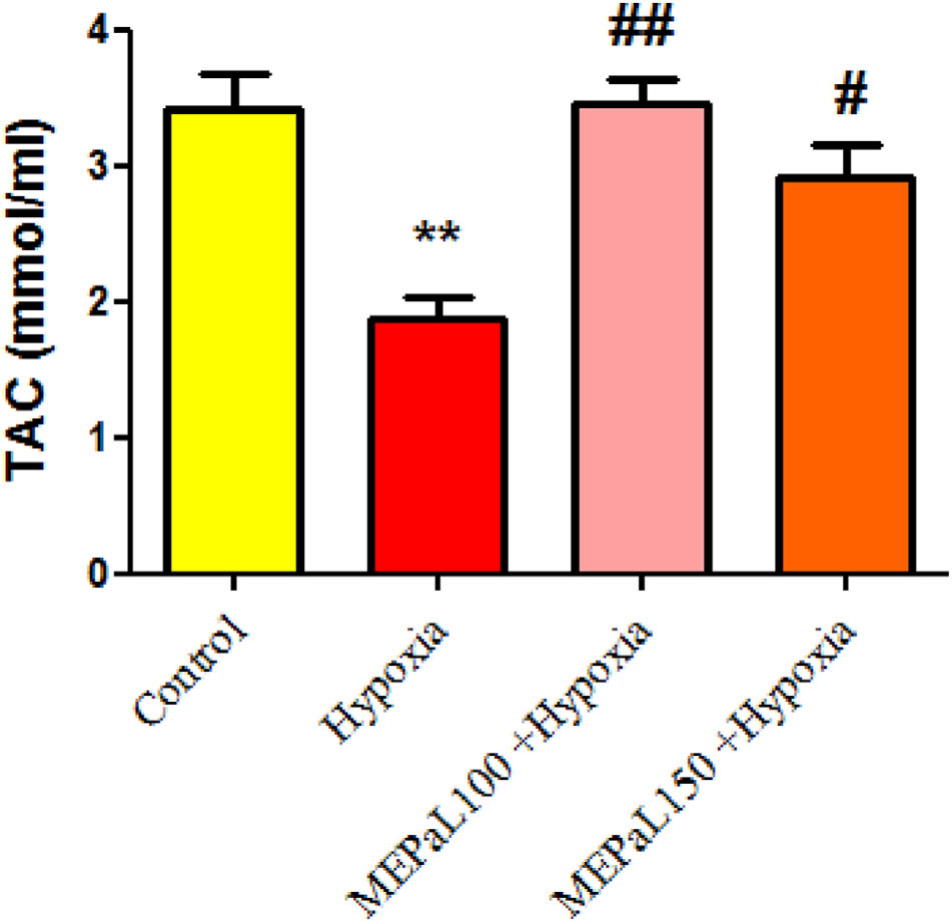
Comparison of total antioxidant capacity (TAC) levels between groups. (**) shows a significant difference at the level of *p* < .01 between the hypoxia and the control groups. (##) (*p* < .01) and (#) (*p* < .05) show a significant difference between the treatment groups with the hypoxia group. All data are represented in mean ± SEM.

The results showed that the level of TAC in the hypoxia group significantly decreased compared to the control group (*p* < .01), whereas in the group exposed to hypoxia + MEPaL at a dose of 100 (*p* < .01) and 150 mg/kg (*p* < .05), it significantly increased compared to the hypoxia group (Figure 11).

#### 3.3.6 Oxidative stress index (OSI)

**FIGURE 12:**
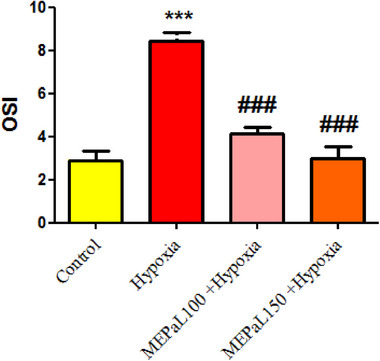
Comparison of oxidative stress index (OSI) levels between groups. (***) shows a significant difference at the level of *p* < .001 between the hypoxia and the control groups. (###) (*p* < .001) shows a significant difference between the treatment groups with the hypoxia group. All data are expressed in mean ± SEM.

The level of OSI in the hypoxia group increased significantly compared to the control group (*p* < .001), whereas, it was significantly decreased in the group exposed to hypoxia + MEPaL at a dose of 100 and 150 mg/kg (*p* < .001) compared to the hypoxia group (Figure 12).

## 4 DISCUSSION

The open‐field test is often used to measure anxiety, exploration, and movement. In this test, the traveled distance and movement speed represent the locomotor performance of the animal. The exploratory behavior was recorded in the open field by measuring the rearing frequency, in which the animal stands on its hind legs to explore their environment. Grooming is a behavior that usually shows increased stress and anxiety in the animal. The results of this research showed that anxiety behavior increased in the hypoxia group compared to the control group, whereas motor activity (traveled distance and movement velocity) decreased in the hypoxia group compared to the control group. Balance was also measured by rotarod test, and a significant decrease was observed in the hypoxia group compared to the control group. In this regard, previous studies have shown that one of the important side effects of pregnancy hypoxia in the long term is a disturbance in the sensory‐motor system.

There are conflicting reports regarding the effect of hypoxia on motor activity and balance. For example, Lubics et al. (2005), in the study of the effect of ischemia/hypoxia on the motor performance of 7‐day‐old mice, observed that the traveled distance, the time spent in corners and walls, and the speed of movement in the open box test were higher in the hypoxia group than in the control group.

Ireland et al. (2011) showed that short‐term acute hypoxia during pregnancy significantly decreased the duration of staying on the rod of rotarod test in the hypoxia compared to the control group. In the open‐field test, the average traveled distance in the hypoxia group was almost 50% less than the control group (Ireland et al., 2009).

It has also been found that hypoxia in neonatal mice leads to motor dysfunction and changes in muscle morphology (Durán‐Carabali et al., 2017).

Clinical evidence also showed sensorimotor system deficit after hypoxia. In another study, the application of hypoxia at the end of pregnancy in rabbits caused movement control disorders and hypertonia in their offspring. The histological results also confirmed the acute impairment in the subcortical motor pathway such as in the thalamus and basal ganglia, after hypoxia (Derrick et al., 2004).

The striatum is one of the important brain structures that is related to movement control and is strongly affected by hypoxia. Studies show that the volume of the striatum decreased by 50% after hypoxia (Hobbs & Oorschot, 2008).

Previous studies have shown damage to the cerebellum of newborns after ischemia–hypoxia, because, at this time, the cerebellum grows rapidly and has high sensitivity and vulnerability to the environment. It is known that cerebellum damage can cause movement disorders and is a possibility of cognitive, behavioral, learning, and emotional dysfunction (Sab et al., 2013).

Oxidative stress following gestational hypoxia is an important mechanism for fetal growth restriction and impaired motor responses after birth (Wan et al., 2021). The results of this research showed that pregnancy hypoxia increased oxidative stress.

Hypoxia reduces energy reserves in nervous tissue and leads to changes such as acidosis, glutamate excitotoxicity, NO production, and oxidative stress in the central nervous system. These biochemical events delay the formation of the nervous system and lead to apoptosis and inflammation (Rodríguez et al., 2020; Wang et al., 2021). The compensatory mechanism following the reduction of oxygen during hypoxia is the increase in mitochondrial activity, which leads to an increase in the production of free radicals (Ni et al., 2021). The production of reactive oxygen and nitrogen species can play a role in the pathogenesis of brain white matter damage and neuronal death, following the activation of astrocytes. Other studies also show an increase in oxidative stress, NO production, and impaired GPx activity in newborn rats born from pregnant mothers exposed to hypoxia (Cannavò et al., 2021; Silvestro et al., 2020). Moreover, the results of this study showed a significant increase in GSH, TOC, and OSI in the hypoxia group compared to the control group, which confirms the increase in oxidative stress. According to these findings, it can be concluded that increased oxidative stress is a possible mechanism for behavior disorder (movement) caused by hypoxia in newborns. It has been proven in various studies that natural antioxidants can play an effective role in protecting neurons and improving memory and cognitive disorders caused by hypoxia (Auti et al., 2022; Ghotbeddin et al., 2021).

The results of a study showed that total oxidant status (TOC) and OSI significantly increase in people with colorectal cancer, and treatment with antioxidants significantly reduces these indicators (Wu et al., 2017). GSH includes two forms: reduced form (GSH) and oxidized form (GSSG), and according to studies, the ratio of GSH/GSSG increases in oxidative stress and the concentration of GSSG decreases, which leads to a decrease in cell resistance against free radicals (Terra et al., 2019; Wu et al., 2017). Recent studies have reported several pharmacological effects of *P. atlantica* such as antimicrobial, antioxidant, antidiabetic, neuroprotective, and antitumor activities (Achili et al., 2020; Rauf et al., 2022). The hydroxyl groups of phenols have the potential to inhibit free radicals, and due to the high amount of phenolic compounds in *Pistacia*, this plant has a high antioxidant capacity in inhibiting the production of free radicals (Hasheminya & Dehghannya, 2020; Tebbi et al., 2023). The results of this study also showed that MEPaL increased movement and improved balance in rats affected by maternal hypoxia compared to the hypoxia group. The concentration of NO in the group receiving 100 mg/kg MEPaL showed a significant decrease compared to the control group, and the doses of 100 and 150 mg/kg MEPaL also increased the concentration of GSH and decreased GSSG compared to the hypoxia group. In addition, doses of 100 and 150 mg/kg MEPaL caused a significant increase in the ratio of GSH to GSSG and decreased OSI and TOC compared to the hypoxia group. In general, the results of this study showed that MEPaL treatment in rats affected by maternal hypoxia improved motor activity, motor coordination, and balance by reducing oxidative stress after hypoxia. Therefore, investigating changes in the expression of inflammatory cytokines and genes related to apoptosis in brain tissue caused by hypoxia is recommended in future studies.

In general, the results of this study showed that MEPaL, by reducing the amount of oxidative stress in neonatal hypoxic, increased movement and improved balance compared to the hypoxic group. Therefore, investigating changes in the expression of inflammatory cytokines and genes related to apoptosis in brain tissue caused by hypoxia is recommended in future studies. Moreover, considering the effective role of MEPaL in reducing oxidative stress caused by hypoxia, its use in clinical trials is suggested.

## AUTHOR CONTRIBUTIONS

Zohreh Ghotbeddin participated in behavior study design and evaluation. She was also responsible for overall supervision. Hadis Nasri contributed to all experimental work and data statistical analysis. Kaveh Rahimi contributed to behavioral study design, MEPaL extract collection, and evaluation. Mohammad Reza Tabandeh participated in the biochemical study design, data collection, and contributed in biochemical parts of the experiment. All the authors reviewed and approved the final manuscript.

## CONFLICT OF INTEREST STATEMENT

All authors have read the journal's authorship agreement and policy on disclosure of potential conflicts of interest. We also certify that the submission is original work and is not under review at any other publication.

### PEER REVIEW

The peer review history for this article is available at https://publons.com/publon/10.1002/brb3.3539.

## Data Availability

The datasets generated for this study are available on request to the corresponding author.
